# Supplementary material for: Synthesis and Biological Evaluation of BODIPY-PF-543
Source: Molecules. 2019 Dec 2;24(23):4408. doi: 10.3390/molecules24234408 (PMC6930633; doi:10.3390/molecules24234408)
Supplement: Supplementary file 1 [file molecules-24-04408-s001.pdf]

Supplementary data

Synthesis and Biological Evaluation of BODIPY-PF-543

Jitendra Shrestha [1†], Gil Tae Hwang [2†], Taeho Lee [3], Seon Woong Kim [1], Yoon Sin Oh [4], Yongseok Kwon [5], Seung Woo Hong [2], Sanghee Kim [6], Hong Seop Moon [1], Dong Jae Baek [1,*], Eun-Young Park [1,*]

[1] College of Pharmacy, Mokpo National University, Jeonnam 58554, Korea; shresthasimon2011@mokpo.ac.kr (J.S.); tjsdnd123@mokpo.ac.kr (S.W.K.); hbsmoon@mokpo.ac.kr (H.S.M.)
[2] Department of Chemistry, Kyungpook National University, Daegu 41566, Korea; giltae@knu.ac.kr (G.T.H.); tmddnzkf1@naver.com (S.W.H.)
[3] College of Pharmacy, Research Institute of Pharmaceutical Sciences, Kyungpook National University, Daegu 41566, Korea; tlee@knu.ac.kr (T.L.)
[4] Department of Food and Nutrition, Eulji University, Seongnam 13135, Korea; ysoh@eulji.ac.kr (Y.S.O.)
[5] Department of Chemistry, Sogang University, Seoul 04107, Korea; ykwon@sogang.ac.kr (Y.K.)
[6] College of Pharmacy, Seoul National University, Seoul 08826, Korea; pennkim@snu.ac.kr (S.K.)
[†] These authors contributed equally to this work

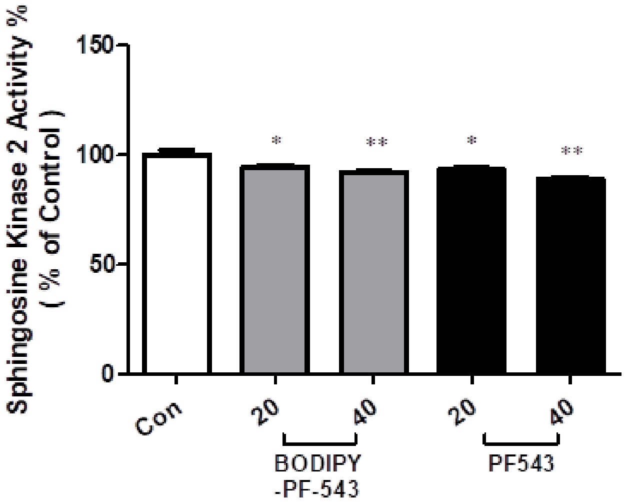

Figure S1. SK2 inhibition assay of BODIPY-PF-543 and PF-543. SK2 activity was measured with 20 μM and 40 μM BODIPY-PF-543 and PF-543 using Echelon's Sphingosine Kinase Activity Assay kit according to the manufacturer's protocol (100 μM sphingosine, 10 μM ATP and 1 ng/μL of recombinant sphingosine kinase 2). Data are means ± SD from three experiments. *p<0.05, **p<0.01 compared with control cells.

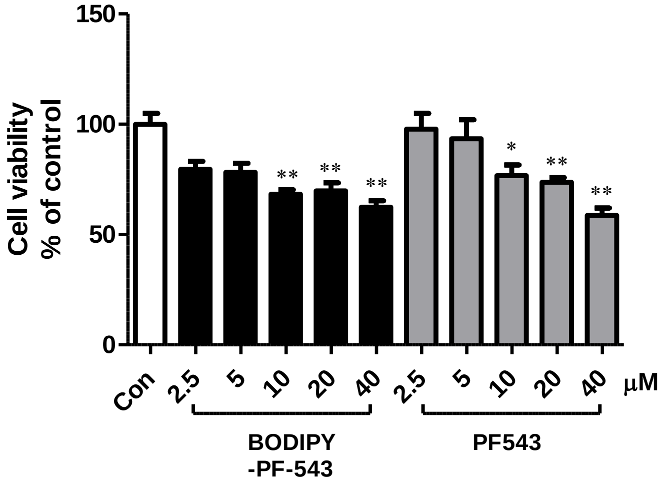

Figure S2. Cell cytotoxic effect of BODIPY-PF-543 and PF-543 in A549 cells. A549 cells were plated in 96 well and treated with BODIPY-PF-543 and PF-543 for 24 h, and cell viability was measured by EZ-CYTOTOX kit. Data are means ± SD (*n*=8). *p<0.05, **p<0.01 compared with control cells.
